# Supplementary figures and images for: Spatiotemporal variation of the maximum cooling effect across edge-to-interior gradients in forest patches of southwestern China
Source: PLoS One. 2026 Feb 12;21(2):e0342179. doi: 10.1371/journal.pone.0342179 (PMC12900313; doi:10.1371/journal.pone.0342179)

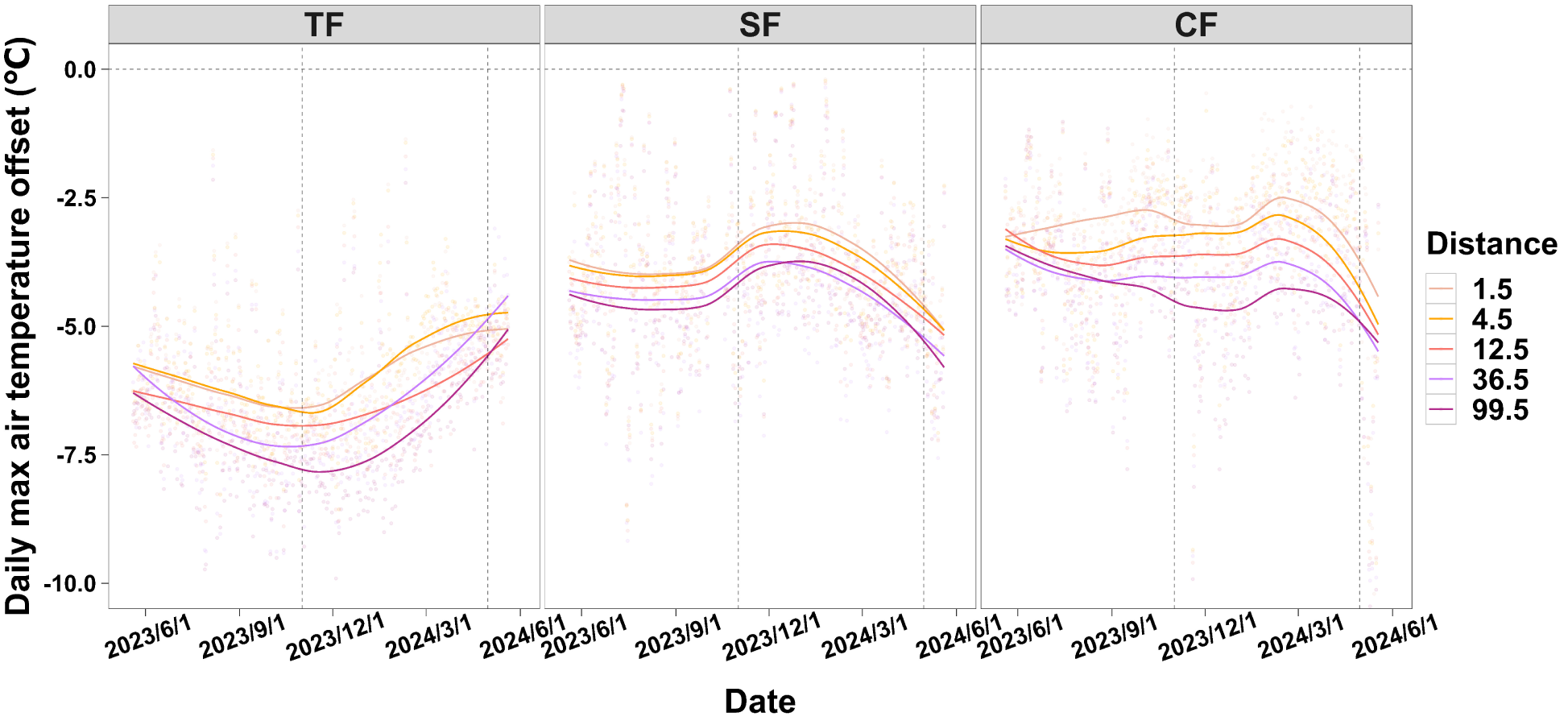

Supplement: S1 Data. — S1 Fig. Spatiotemporal sequence chart of daily MCI (5th percentile of daytime hourly temperature offsets) in natural forest types. The lines show the daily variation trend of MCI. Different panels represent natural forest type in different climatic zones. The colors of the lines and dots show the distance from the edge. Slight jittering has been applied along the X-axis to improve clarity. S2 Fig in S1 Data. Predictions of robust maximum cooling (MCI, C) as function of the distance to the edge (m). The lines show model predictions of significant interaction between forest ecosystem types and edge distance. The colors of the lines and points show natural forest type in different climatic zones. Slight jittering has been applied along the X-axis to improve clarity. S3 Fig in S1 Data. Spatial and temporal comparison of robust maximum cooling (MCI) in the decoupled interior and coupled edge zones of natural forest types at monthly and seasonal scales. Figure a is monthly scale, figure b is seasonal scale. Different panels represent natural forest type in different climatic zones. Comparison of maximum cooling between coupled and decoupled zones is represented by lowercase letters, while differences among forest types across months and seasons are represented by uppercase letters. The horizontal line in a box plot represents the median of the data, while the box limits indicate the interquartile range, extending to the minimum and maximum values. S4 Fig in S1 Data. Model predictions of the air-temperature offset (C) as a function of distance from the forest edge (m), combining the original automatic monitoring data and the new snapshot-transect measurements. Colored lines indicate natural forest types in different climatic zones (CF = temperate coniferous forest, SF = subtropical evergreen broadleaf forest, TF = tropical forest). Gray shaded ribbons show 95% confidence intervals. Point transparency and jittering were applied to improve visibility. S1 Table. GAM results f [file pone.0342179.s001.zip › Supporting Information - CompressedZIP File Archive/S1 Fig.tif]

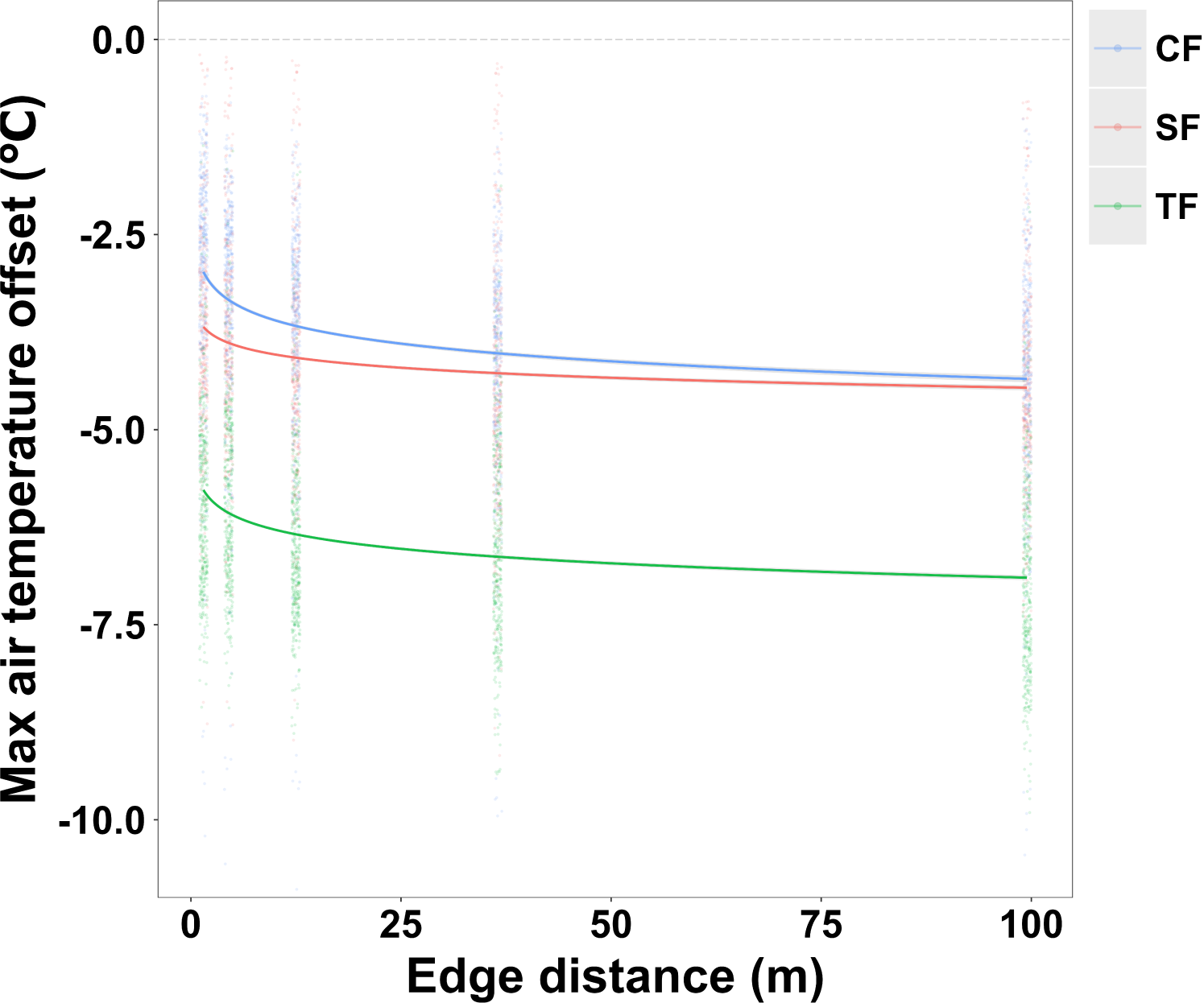

Supplement: S1 Data. — S1 Fig. Spatiotemporal sequence chart of daily MCI (5th percentile of daytime hourly temperature offsets) in natural forest types. The lines show the daily variation trend of MCI. Different panels represent natural forest type in different climatic zones. The colors of the lines and dots show the distance from the edge. Slight jittering has been applied along the X-axis to improve clarity. S2 Fig in S1 Data. Predictions of robust maximum cooling (MCI, C) as function of the distance to the edge (m). The lines show model predictions of significant interaction between forest ecosystem types and edge distance. The colors of the lines and points show natural forest type in different climatic zones. Slight jittering has been applied along the X-axis to improve clarity. S3 Fig in S1 Data. Spatial and temporal comparison of robust maximum cooling (MCI) in the decoupled interior and coupled edge zones of natural forest types at monthly and seasonal scales. Figure a is monthly scale, figure b is seasonal scale. Different panels represent natural forest type in different climatic zones. Comparison of maximum cooling between coupled and decoupled zones is represented by lowercase letters, while differences among forest types across months and seasons are represented by uppercase letters. The horizontal line in a box plot represents the median of the data, while the box limits indicate the interquartile range, extending to the minimum and maximum values. S4 Fig in S1 Data. Model predictions of the air-temperature offset (C) as a function of distance from the forest edge (m), combining the original automatic monitoring data and the new snapshot-transect measurements. Colored lines indicate natural forest types in different climatic zones (CF = temperate coniferous forest, SF = subtropical evergreen broadleaf forest, TF = tropical forest). Gray shaded ribbons show 95% confidence intervals. Point transparency and jittering were applied to improve visibility. S1 Table. GAM results f [file pone.0342179.s001.zip › Supporting Information - CompressedZIP File Archive/S2 Fig.tif]

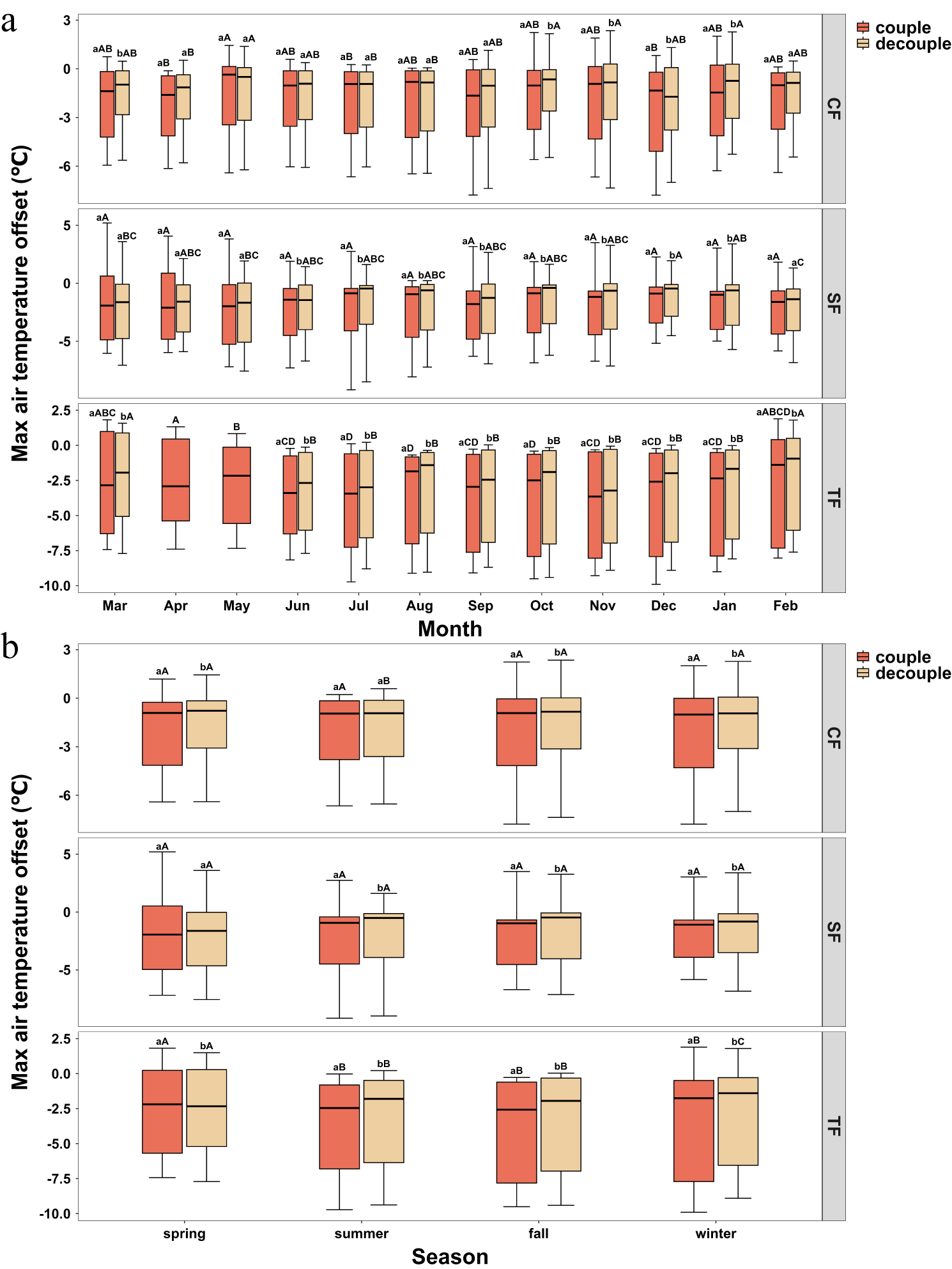

Supplement: S1 Data. — S1 Fig. Spatiotemporal sequence chart of daily MCI (5th percentile of daytime hourly temperature offsets) in natural forest types. The lines show the daily variation trend of MCI. Different panels represent natural forest type in different climatic zones. The colors of the lines and dots show the distance from the edge. Slight jittering has been applied along the X-axis to improve clarity. S2 Fig in S1 Data. Predictions of robust maximum cooling (MCI, C) as function of the distance to the edge (m). The lines show model predictions of significant interaction between forest ecosystem types and edge distance. The colors of the lines and points show natural forest type in different climatic zones. Slight jittering has been applied along the X-axis to improve clarity. S3 Fig in S1 Data. Spatial and temporal comparison of robust maximum cooling (MCI) in the decoupled interior and coupled edge zones of natural forest types at monthly and seasonal scales. Figure a is monthly scale, figure b is seasonal scale. Different panels represent natural forest type in different climatic zones. Comparison of maximum cooling between coupled and decoupled zones is represented by lowercase letters, while differences among forest types across months and seasons are represented by uppercase letters. The horizontal line in a box plot represents the median of the data, while the box limits indicate the interquartile range, extending to the minimum and maximum values. S4 Fig in S1 Data. Model predictions of the air-temperature offset (C) as a function of distance from the forest edge (m), combining the original automatic monitoring data and the new snapshot-transect measurements. Colored lines indicate natural forest types in different climatic zones (CF = temperate coniferous forest, SF = subtropical evergreen broadleaf forest, TF = tropical forest). Gray shaded ribbons show 95% confidence intervals. Point transparency and jittering were applied to improve visibility. S1 Table. GAM results f [file pone.0342179.s001.zip › Supporting Information - CompressedZIP File Archive/S3 Fig.tif]

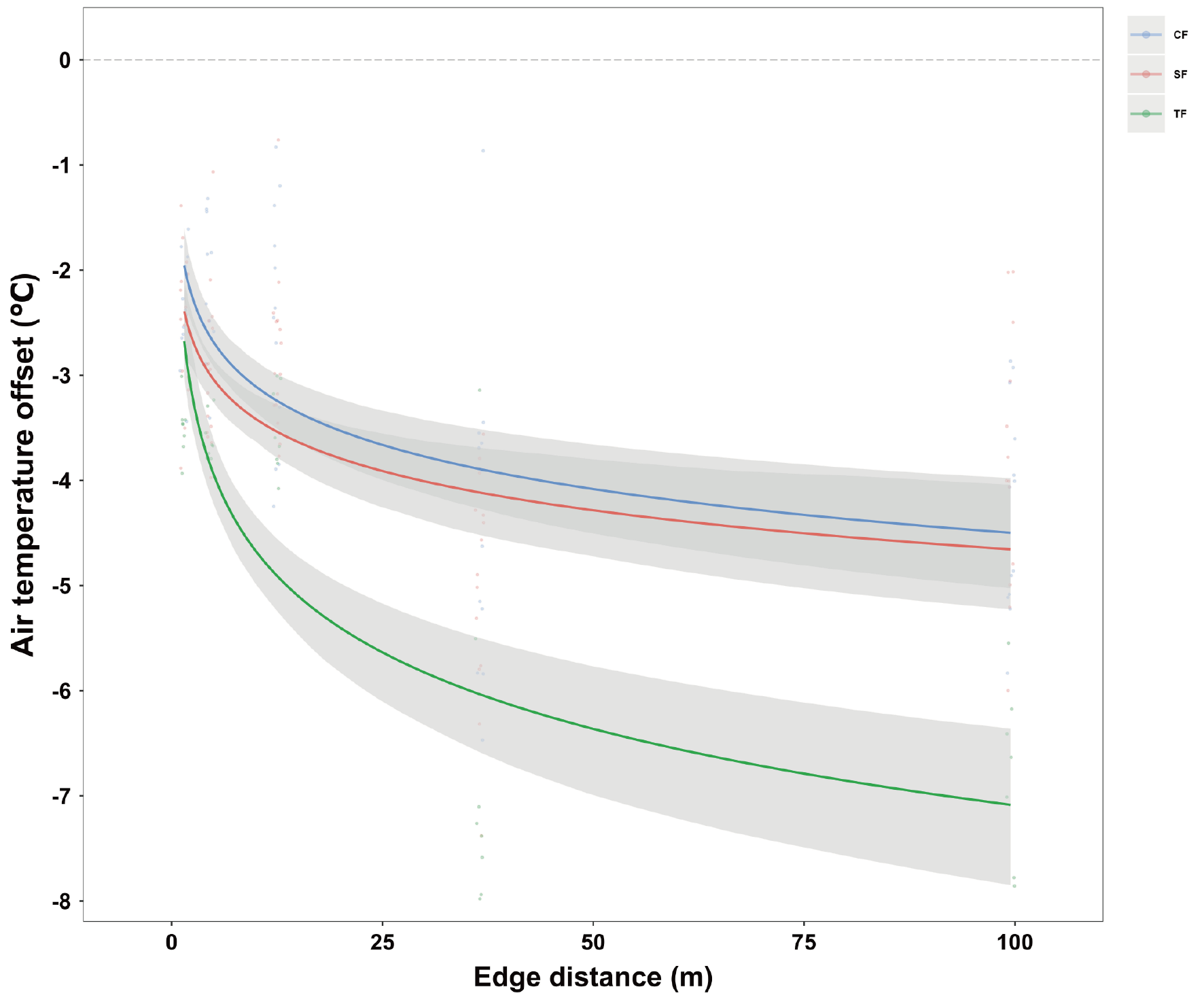

Supplement: S1 Data. — S1 Fig. Spatiotemporal sequence chart of daily MCI (5th percentile of daytime hourly temperature offsets) in natural forest types. The lines show the daily variation trend of MCI. Different panels represent natural forest type in different climatic zones. The colors of the lines and dots show the distance from the edge. Slight jittering has been applied along the X-axis to improve clarity. S2 Fig in S1 Data. Predictions of robust maximum cooling (MCI, C) as function of the distance to the edge (m). The lines show model predictions of significant interaction between forest ecosystem types and edge distance. The colors of the lines and points show natural forest type in different climatic zones. Slight jittering has been applied along the X-axis to improve clarity. S3 Fig in S1 Data. Spatial and temporal comparison of robust maximum cooling (MCI) in the decoupled interior and coupled edge zones of natural forest types at monthly and seasonal scales. Figure a is monthly scale, figure b is seasonal scale. Different panels represent natural forest type in different climatic zones. Comparison of maximum cooling between coupled and decoupled zones is represented by lowercase letters, while differences among forest types across months and seasons are represented by uppercase letters. The horizontal line in a box plot represents the median of the data, while the box limits indicate the interquartile range, extending to the minimum and maximum values. S4 Fig in S1 Data. Model predictions of the air-temperature offset (C) as a function of distance from the forest edge (m), combining the original automatic monitoring data and the new snapshot-transect measurements. Colored lines indicate natural forest types in different climatic zones (CF = temperate coniferous forest, SF = subtropical evergreen broadleaf forest, TF = tropical forest). Gray shaded ribbons show 95% confidence intervals. Point transparency and jittering were applied to improve visibility. S1 Table. GAM results f [file pone.0342179.s001.zip › Supporting Information - CompressedZIP File Archive/S4 Fig.tif]
