# Supplementary material for: Spatiotemporal variation of the maximum cooling effect across edge-to-interior gradients in forest patches of southwestern China
Source: PLoS One. 2026 Feb 12;21(2):e0342179. doi: 10.1371/journal.pone.0342179 (PMC12900313; doi:10.1371/journal.pone.0342179)
Supplement: Attachment — Submitted filename: PONE-D-25-08797_Review_v1.docx [file pone.0342179.s002.docx]

Spatiotemporal variation of the Maximum Cooling Effect across Edge-to-Interior Gradients in Natural Forest Patches of Southwest China

**Summary:**

This manuscript investigates how ambient air temperature changes along a gradient of forest to non-forested. The manuscript is well prepared, simple, and concisely written. However, the authors should work on integrating how this work contributes to ecological theory i.e., niche dynamics, community assembly processes, tension-facilitation hypothesis for buffering climate change effects. A major limitation of the manuscript is the lack of replication in transects between ecosystem types. As is, the authors are drawing inference for entire ecosystems based on 1 transect and 5 plots per transect. Moreover, the authors compare non-forest temperatures derived from 1 plot/data point. The study design is concerning that there needs to be more replication in transects and non-forested ecosystems should have comparable transect designs to account for variability if it exists. The authors should add a section in the Discussion to discuss the limitations of their study and to be transparent in how these limitations may affect their interpretation of these data/results. This section should also acknowledge how to design more appropriate sampling strategy to test the identified hypothesis. They should also add the number of “n” in the Methods for upfront transparency.

**Major Issues:**

Lack of transect replication is a major limitation for drawing statistical inference and the interpretation of ecosystem level processes.

**Minor Issues:**

**Abstract:**

Line 25-26: More specifically, I think this highlights how critical it is to retain patch sizes and shapes that allow for interior forest conditions as edges are more vulnerable to the effects of climate change.

**Introduction:**

Line 96-98: This is a good point but on the other end of this is knowing how much interior forest exists to support biodiversity where under canopy climate is more conceivably more stable. This requires consideration of patch size and shape post edge assessment.

Line 102-105: Does this study consider DEI of different forest by age within the matrix of forest landscapes? Not only forest age but edge orientation, i.e., aspect, slope, elevation? These are crucial factors that influence solar radiation, intensity, and duration across temporal dimensions.

**Methods:**

Line 128: Latitudinal lines on the map would you be useful to demonstrate some of points mentioned in the Discussion. Also add TF, SF, and CF codes to map to parallel the presentation of results in the manuscript.

Line 130: The numbers are hard to read on panel b; also 5 samples plot, irregularly spaced over a 100 meter transect likely does not capture the gradient effect temperature from edge to interior – the large gap between plot 3 and plot 4 and between plot 4 and plot 5 does not allow for fine scale or microclimate detection of an asymptotic relationship of temperature and distance to interior. Having regular space plots/data stations would allow for the detection of inflection points at a fine scale.

Line 133: How many transects per forest type? Line 139: was this only 1 transfect per forest type and thus 5 plots per forest type? If so, this is a fairly low number of plots to draw conclusions on temperature gradients for a given forest type where structural variability likely differs greatly as well as edge orientation as I mentioned above.

Line 158: “these data….. were collected”

**Results:**

Line 229: Remove double label on Fig 2.

Line 229: A gray background around the trend line would show the variability in data more easily than the spread of data points being used – which is hard to see given the size.

Line 229: The authors could change the order to CF, SF, and TF to follow North to South patterns of ecosystems show in Fig 1. Although the current order follows the general trend i.e., Line 298 of increased DEI cooling across ecosystem types.

**Discussion:**

Line 284: Discussion on how the evidence/results support your hypothesis and how this work contributes to ecological theory would be welcomed.

Line 284: Integrating plot level measures of structural variability i.e., species abundance, canopy closure would be a nice addition to testing some local scale effects of edge-to-interior temperature buffering.

Line 325: Perhaps the “effect of edges” is a better description than “content”.

Line 330: Is this really a reduction in spatial scales, rather a smaller gradient of change.

Line 337-339: There are regional (landscape context) and latitudinal differences as well as local scale differences due to forest canopy snow interception that reduces soil moisture content. Expanding the discussion to include these points would be welcomed.

Line 346: The paragraph jumps around quite a bit. The topic sentence is “edges effects define interior forests conditions” however the paragraph unpacks buffering effects, regional effects, and seasonal effects of edges with little discussion of how these conditions influence or define interior forest conditions.

**Conclusions:**

Line 349: “uses”

Line 359: A discussion on species which may use patch edges for different life history or seasonal functions would improve the characterization of forest variability and shape how we understand within patch variability. The current emphasis is on temperature sensitive species, but some species may need warmer edges where different plants grow for nutritional reasons, for example. Thus, less buffering may increase within patch biodiversity via temperature filtering for different species.

Line 367: May also be useful for conducting connectivity analysis either species agnostic or species specific by integrating local scale temperature habitat selection.

Line 369: the number of transects is not clearly defined and is a major concern for interpretation of results and their meaningfulness to ecological theory and land management. Line 139 reads as if only 1 transect with 5 plots per transect was established for each of the ecosystem types. This is a major limitation given the lack of replication.

**Recommendation:**

Major revisions
