# Supplementary material for: Spatiotemporal variation of the maximum cooling effect across edge-to-interior gradients in forest patches of southwestern China
Source: PLoS One. 2026 Feb 12;21(2):e0342179. doi: 10.1371/journal.pone.0342179 (PMC12900313; doi:10.1371/journal.pone.0342179)
Supplement: Attachment — Submitted filename: XZJ_Response to Reviewer4.docx [file pone.0342179.s005.docx]

Spatiotemporal variation of the Maximum Cooling Effect across Edge-to-Interior Gradients in Natural Forest Patches of Southwest China

Reviewer #4:

It was a pleasure to review the manuscript “Spatiotemporal variation of the Maximum Cooling Effect across Edge-to-Interior Gradients in Natural Forest Patches of Southwest China” for PLOS One. In this manuscript, authors examined the spatiotemporal dynamics of maximum cooling at forest edges across three forest types in Southwest China. The study has strong relevance, particularly given increasing interest in how forest microclimates buffer against global climate change. Results clearly show that maximum cooling and DEI vary by forest type and season, with warmer forests exhibiting stronger cooling but smaller DEI. The findings are well contextualized with existing global studies. I think this work has potential for publication, especially as it tackles an understudied topic, edge-related microclimate variability but I will leave the decision on fit and scope to the editor. Especially since I am not an expert on this topic. So most of the issues I raised are statistical/inference concerns and I would recommend that this manuscript being peer reviewed by an expert on forest microclimates.

My main concern is with clarity around the methods and the framing of novelty. While the abstract mentions transect-based in situ monitoring, it lacks detail on spatial replication, sensor placement, or data resolution, which are critical to interpreting spatiotemporal patterns. Also, terms like “maximum cooling” need more precise definition—does this refer to mean maximum difference in temperature between edge and interior, or some peak event? Without these clarifications, the magnitude and implications of their findings are hard to judge.

The logic and novelty of comparing different forest types (temperate, subtropical, and tropical) are compelling, but the manuscript does not clearly articulate how these comparisons inform theory or practice beyond the descriptive level.

I like the flow of the manuscript, it has great coherence and cohesion, and it makes the readers enthusiastic about following the manuscript and continue reading it.

Once methodological clarity is improved and the authors better articulate their conceptual contributions, I would be happy to provide more detailed comments on a full version of the manuscript.

Response: Thank you for the reviewers’ guiding suggestions. To improve the clarity and transparency of our methodology, we have made several revisions throughout the manuscript. In **Section 2.2** (**lines 156–159**), we added details on sensor placement; at **lines 162–164**, we described the data resolution and quantity. In **Section 2.3.1** (**lines 180–184)**, **2.3.2 (lines 199–200)**, and **2.3.3 (lines 210–213)**, we provided additional information on the analysis of edge microclimate gradient variation and clarified the data and sample sizes used in the DEI analysis. The calculation procedure for maximum cooling has been reorganized in **Section 2.3.4** (**lines 227–235**), and its definition and application have been clarified in the **Results Section** (**lines** **241–243**).

To further enhance the interpretation of our findings, we expanded the discussion on the ecological significance of forest edge temperature effects across climatic zones, and on the role and dynamics of DEI. Specifically, in **Sections 4.1** (**lines 352–364**) and **4.2** (**lines 390–396**), we connected our findings to ecological theory, including concepts of niche stability, forest structural complexity, and the influence of edge microclimates on species distributions. Additionally, we acknowledge the importance of spatial replication in capturing spatiotemporal microclimate patterns. A new **Section** (**4.3**, **lines 400–425**) has been added to the Discussion, where we address this limitation and propose future directions using more spatially robust sampling frameworks.

**Material and methods Section 2.2**, lines **156–159:**

A temperature sensor (HOBO® MX2301A) was mounted at 1.5m above the ground on each pole (Fig 1c), with a protective PVC pipe (diameter: 100mm, height: 10cm) shield to prevent direct sunlight exposure and thereby reduce the influence of solar radiation on temperature measurements [44].

**Material and methods Section 2.2**, lines **162-164：**

The monitoring interval for each sensor was set to 10 minutes, with data collected from May 2023 to May 2024 (n = 52,560 paired observations, based on 365 days per year, 24 hours per day, and 6 measurements per hour).

**Material and methods Section 2.3.1**, lines **180–184:**

To assess the maximum temperature buffering effect from forest edge to interior, the spatial dynamics of edge influence distance, and the most stable and intense cooling conditions across forest types, the 5th percentile of hourly temperatures (n1 = 8760) and the 5th percentile of daily temperature offsets (n2 = 365) were calculated for each sensor and monitoring position respectively, during daytime hours.

**Material and methods Section 2.3.2**, lines **199–200:**

These models were consistently applied to analyze edge gradient variations at annual (n = 365 per plot), seasonal (n = 90–93 per plot), and monthly (n = 30–31 per plot) time scales.

**Material and methods Section 2.3.3**, lines **210–213:**

The slope, representing the daily buffering effect for each temperature sensor within the forest, was calculated and grouped at three temporal scales—annually (n = 365 per plot), seasonally (n = 90–93 per plot), and monthly (n = 30–31 per plot).

**Material and methods Section 2.3.4**, lines **227–235:**

In this study, forest zones from the edge (0 m) up to the DEI were defined as coupled with the macroclimate, while zones beyond the DEI were considered statistically decoupled. Using daily 5th percentile temperature offset data, the maximum cooling effect—defined as the greatest observed difference between forest and macroclimate temperatures—was calculated separately within the coupled and decoupled zones for each forest type and temporal scale (monthly, seasonal, and annual).

The Kruskal–Wallis non-parametric test, which is appropriate for non-normally distributed data, was employed to compare the magnitudes of maximum cooling between zones and among forest types. Through this analysis, it was determined whether maximum cooling differed significantly between coupled and decoupled zones and across forest types.

**Results Section 4.1**, lines **241-243:**

Over the course of the year, the maximum air temperature offset from forest edge to interior was −6.3 ± 1.3 °C in tropical forests, −4.1 ± 1.4 °C in subtropical forests, and −3.7 ± 1.5 °C in temperate forests (S2 Fig)

**Discussion Section 4.1**, lines **352-364:**

These findings highlight the diverse thermal tolerances of plant species across climatic zones [57], with differential heat tolerance influencing habitat selection and driving distributional shifts toward warmer microclimates [58]. Microclimates delineate the realized thermal niches of forest plant species [59]. As microclimatic temperatures decline toward forest edges, species with varying thermal requirements across life stages may benefit [60], shaping their thermal preferences and habitat selection. These processes critically influence species composition and spatial distribution, as observed in birds [61] and insects [62,63]. Overall, our findings support the hypothesis that forest edges across climatic gradients exhibit distinct cooling patterns. The temperature offset in forests across different climatic zones was markedly distinct, with edge areas exhibiting increased cooling in warmer regions. The cooling thresholds for TF, SF, and CF were 8.2°C, 7.0°C, and 5.8°C, respectively. Edge-induced temperature effects vary across space and seasons among forest types along climatic gradients. These findings align with the ecological niche dynamics theory, suggesting that species persistence is shaped by localized thermal environments within broader ecological contexts. **Discussion Section 4.2**, lines **391-397:**

Identifying the minimum DEI gives an indication of the critical forest buffer zone required for the survival of temperature-sensitive understory species across TF, SF and CF [18,34,19,48,73]. Through monthly assessments of DEI, we revealed pronounced seasonal and regional variations, highlighting how forest types and climatic regimes shape the spatial extent of edge-induced microclimatic shifts. These findings suggest that edge-mediated microenvironments may function as ecological filters, influencing community assembly processes and supporting the identification of microrefugia, particularly for temperature-sensitive species [59,74].

Main comments:

- Authors claim to support existing hypotheses, but they don't indicate what those hypotheses are. It would help to be more specific about how these findings advance or challenge current understanding.

Response: Thank you for your valuable feedback. In the revised manuscript, we have reorganized the hypothesis statement in the **Introduction** (**lines 116–119**) to improve clarity and logical flow. Additionally, in the Discussion section, we have strengthened the theoretical interpretation of our findings. Specifically, in **Section 4.1** (**lines 352–364**), we elaborate on how our results link temperature buffering to ecological theory, including concepts of niche stability, forest structural complexity, and the influence of edge microclimates on species distribution. In **Section 4.2** (**lines** **391–397**), we clarify the ecological significance of the Distance of Edge Influence (DEI) and relate its dynamic temporal pattern to the spatial maintenance of thermal conditions within forests. Finally, we emphasize the relevance of DEI for delineating forest microclimates in the context of biodiversity conservation and landscape management.

**Introduction Section**, lines **116–119:**

We hypothesize that (1) forest cooling intensifies from edge to interior across all temporal scales; (2) forests in warmer regions exhibit shorter DEI and greater maximum cooling; and (3) temperature offsets and DEI fluctuate seasonally, with both reaching their extremes at the monthly scale.

**Discussion Section 4.1**, lines **352-364:**

These findings highlight the diverse thermal tolerances of plant species across climatic zones [57], with differential heat tolerance influencing habitat selection and driving distributional shifts toward warmer microclimates [58]. Microclimates delineate the realized thermal niches of forest plant species [59]. As microclimatic temperatures decline toward forest edges, species with varying thermal requirements across life stages may benefit [60], shaping their thermal preferences and habitat selection. These processes critically influence species composition and spatial distribution, as observed in birds [61] and insects [62,63]. Overall, our findings support the hypothesis that forest edges across climatic gradients exhibit distinct cooling patterns. The temperature offset in forests across different climatic zones was markedly distinct, with edge areas exhibiting increased cooling in warmer regions. The cooling thresholds for TF, SF, and CF were 8.2°C, 7.0°C, and 5.8°C, respectively. Edge-induced temperature effects vary across space and seasons among forest types along climatic gradients. These findings align with the ecological niche dynamics theory, suggesting that species persistence is shaped by localized thermal environments within broader ecological contexts. **Discussion Section 4.2**, lines **391-397:**

Identifying the minimum DEI gives an indication of the critical forest buffer zone required for the survival of temperature-sensitive understory species across TF, SF and CF [18,34,19,48,73]. Through monthly assessments of DEI, we revealed pronounced seasonal and regional variations, highlighting how forest types and climatic regimes shape the spatial extent of edge-induced microclimatic shifts. These findings suggest that edge-mediated microenvironments may function as ecological filters, influencing community assembly processes and supporting the identification of microrefugia, particularly for temperature-sensitive species [59,74].

- The manuscript could better characterize the nature of forest edges (e.g., abruptness, adjacent land use) and discuss how edge contrast might influence microclimate gradients.

Response: Thank you for the constructive feedback from the reviewers. We acknowledge that factors such as forest edge contrast (i.e., adjacent land-use types), edge orientation, slope, aspect, and forest age can influence microclimatic gradients. Accordingly, in the revised manuscript, we have added a description of forest age in **Section 2.1** (**lines 138–139**) and clarified how environmental variables were controlled during transect selection in **Section 2.2** (**lines 147–150**). Furthermore, in the newly added **Section 4.3** of the **Discussion** (**lines 400-425**), we propose a more comprehensive future research framework. Specifically, we recommend adopting a stratified and replicated design across topographic and structural gradients to improve the robustness and generalizability of ecological inferences.

**Material and methods Section 2.1**, lines **138-139:**

The forests have remained ecologically stable for decades, with minimal anthropogenic impact and no recent natural disturbances.

**Material and methods Section 2.2**, lines **147-150:**

Transects were established at the border between forested and non-forested areas, all oriented towards the south, southwest, or southeast. The selected forest area was large enough on all sides to allow for the dissipation of macroclimatic effects, ensuring that the distance from the forest interior to any non-forested patches exceeded 100 meters [41].

**Discussion Section 4.3**, lines **401-426:**

One major limitation of this study is that only a single transect was established for each forest type. While long-term microclimatic monitoring and consistent observational methods were employed to enhance the reliability of the data, this design constrains the spatial representativeness and generalizability of the findings. Moreover, the forests sampled were relatively undisturbed and mature, limiting the applicability of the results to regenerating or disturbed forests. Future studies should incorporate multiple transects per forest type, ideally spanning different stand ages and successional stages [65,66], to assess how edge effects vary across forest development trajectories. To improve the spatial resolution of edge effect estimations, we recommend establishing replicated transects both horizontally and vertically within forests [7,14,75]. Additionally, plot spacing should follow a combination of equidistant and logarithmic intervals to better capture non-linear gradients and potential inflection points of microclimatic buffering. Including multiple transects in forests with contrasting edge orientations, slope gradients, and altitudinal zones will also allow for a more robust evaluation of how edge microclimates interact with terrain and climatic variation. Beyond the local scale, future studies should incorporate the landscape configuration—specifically the size and shape of forest patches—to evaluate the extent of interior forest microclimate buffering. Although our study focused on temperature dynamics, integrating plot-level vegetation structure (e.g., canopy closure, understory density, species composition) [3,26], topographic variables (e.g., elevation, aspect, slope) [76], and macroclimatic background (e.g., annual temperature and precipitation) [7] could substantially improve our understanding of temperature buffering mechanisms from edge to interior, thereby strengthening the explanatory power of edge effect models. Furthermore, linking edge temperature gradients to species habitat use and functional diversity will help contextualize the ecological significance of DEI. Some species, especially thermophilic or generalist taxa, may benefit from warmer edge zones, using them for feeding or seasonal activities [77,78]. Therefore, less buffering may not always indicate ecological degradation; instead, it may increase within-patch biodiversity through temperature niche partitioning. Future work should explore how edge microclimates filter community composition and influence habitat connectivity, which may inform both species-specific and species-agnostic conservation strategies.

- The study relies on a limited number of transects per forest type, which may restrict the generalizability of the findings. Future work should increase spatial replication to account for within-type variability.

Response: Thank you for the constructive feedback from the reviewers. We fully acknowledge the limitations regarding the spatial replication of transects and their potential influence on the study outcomes. In response, we have added a new section to the **Discussion—Section 4.3**, "Major limitations and recommendations for future research" (**lines 401–426**)—to address this issue. There, we explicitly discuss how the number of transects may affect the spatial representativeness and interpretation of the results.

To improve future study designs, we propose adopting more comprehensive approaches, including increased spatial replication across topographic and structural gradients. In future research, we plan to enhance transect repeatability to strengthen the generalizability and ecological inference of our findings. Nevertheless, we believe the transects selected in this study are highly representative, and the temporal replication of data collection adds further robustness to the conclusions. Thus, despite limitations, the results still offer valuable insights into edge microclimate dynamics and forest thermal buffering processes.

**Discussion Section 4.3**, lines **401-426:**

One major limitation of this study is that only a single transect was established for each forest type. While long-term microclimatic monitoring and consistent observational methods were employed to enhance the reliability of the data, this design constrains the spatial representativeness and generalizability of the findings. Moreover, the forests sampled were relatively undisturbed and mature, limiting the applicability of the results to regenerating or disturbed forests. Future studies should incorporate multiple transects per forest type, ideally spanning different stand ages and successional stages [65,66], to assess how edge effects vary across forest development trajectories. To improve the spatial resolution of edge effect estimations, we recommend establishing replicated transects both horizontally and vertically within forests [7,14,75]. Additionally, plot spacing should follow a combination of equidistant and logarithmic intervals to better capture non-linear gradients and potential inflection points of microclimatic buffering. Including multiple transects in forests with contrasting edge orientations, slope gradients, and altitudinal zones will also allow for a more robust evaluation of how edge microclimates interact with terrain and climatic variation. Beyond the local scale, future studies should incorporate the landscape configuration—specifically the size and shape of forest patches—to evaluate the extent of interior forest microclimate buffering. Although our study focused on temperature dynamics, integrating plot-level vegetation structure (e.g., canopy closure, understory density, species composition) [3,26], topographic variables (e.g., elevation, aspect, slope) [76], and macroclimatic background (e.g., annual temperature and precipitation) [7] could substantially improve our understanding of temperature buffering mechanisms from edge to interior, thereby strengthening the explanatory power of edge effect models. Furthermore, linking edge temperature gradients to species habitat use and functional diversity will help contextualize the ecological significance of DEI. Some species, especially thermophilic or generalist taxa, may benefit from warmer edge zones, using them for feeding or seasonal activities [77,78]. Therefore, less buffering may not always indicate ecological degradation; instead, it may increase within-patch biodiversity through temperature niche partitioning. Future work should explore how edge microclimates filter community composition and influence habitat connectivity, which may inform both species-specific and species-agnostic conservation strategies.

- The authors state that they used a stepwise procedure to remove non-significant effects. Stepwise model selection (particularly automated approaches) is widely criticized for inflating Type I error, ignoring model uncertainty, and producing biased coefficient estimates. It’s often not recommended unless justified (e.g., via information-theoretic criteria like AIC/BIC and cross-validation). Therefore, authors should clarify whether this was based on AIC, BIC, or p-values, and ideally use model comparison techniques grounded in model selection theory (e.g., multimodel inference or model averaging).

Response: The reviewer’s suggestions are critical, and indeed we overlooked these issues in our previous analysis. After re-examining the methods used, we found that they employed backward steps, using likelihood ratio test for random effects and F-tests for fixed effects [1]. This approach clearly increases the probability of Type Ⅱ errors [2]. However, in this study, such an impact should be negligible. Firstly, the settings for both fixed and random effects are based on the analysis by Meeussen et al. [3], which have been well-applied and tested. We used the step method primarily to verify if the setting of random effects was reasonable. Given that our study only uses a small number of variables with very weak multicollinearity among them, the Type Ⅱ error of the model would not accumulate to an exaggerated extent. However, such concerns still exist, so we conducted supplementary comparisons on all possible models and used AIC as the criterion for evaluation. We found that there were no significant differences in AIC values between several models (Table S1). Therefore, we opted for the simplest m2 model, which is consistent with the model previously used by us. Additionally, we have included a more detailed explanation of the variable selection process in the revised manuscript, specifically in the **Materials and Methods** section (**Section 2.3.2, lines 194–197**).

| Table S1. Comparison of four random effects models. | | | | | |
| --- | --- | --- | --- | --- | --- |
|  | n | AIC | BIC | logLik | Random |
| m1 | 8 | 51562 | 51620 | -25773 | ~ 1 \| DateDay:(Region:Plot) |
| m2 | 8 | 51562 | 51620 | -25773 | ~ 1 \| DateDay:Region |
| m3 | 10 | 51566 | 51639 | -25773 | ~ 1 \| Region/ DateDay |
| m4 | 11 | 51568 | 51648 | -25773 | ~ 1 \| Site:DateDay + 1 \| Region/Plot |

**Materials and Methods Section 2.3.2**, lines **194–197:**

We compared several combinations of random effects based on AIC and selected the simplest model with the lower AIC. Finally, a random intercept with date nested within regions was determined. For the robustness of the models, all fixed effects were retained, even if they are not significant

- Daily temperature data was analyzed using linear mixed models, but there's no mention of controlling for temporal autocorrelation. Repeated daily measurements at the same locations can violate the assumption of independence, which may inflate Type I error or bias standard errors. Authors should consider including temporal autocorrelation structures or explicitly discuss how they addressed this issue.

Response: Spurious regression is quite common in time-series correlation analysis [4]. However, in linear mixed models, this phenomenon should be discussed separately. In our study, the fixed effects involved, including distance and location, do not change over time; therefore, time autocorrelation only affects the estimation of random effects, which is not the focus of our analysis. As pointed out by Davis [5]: “Although AIC improves with the addition of serially correlated error, it does not necessarily follow that estimators of the fixed effects will have lower standard errors (and since the estimators of the fixed effects are unbiased, mean squared error) by including rho (AR (1) model).” This suggests that removing effect of time autocorrelation is not necessary in our analysis.

However, we still conducted an analysis of autocorrelation. We set up four models; two considered time autocorrelation while the other two did not. Additionally, since we used different R packages (lme4 cannot set a non-identity covariance matrix for residuals, so we used “nlme”), we also set up comparisons with the original methods. Generally, it is usually adequate to assume a first-order autocorrelated structure [6], so we set an AR (1) model to remove the impact of time autocorrelation. We configured five models (Table S2), where m1 sets simple autocorrelation by following sampling order; m2 sets full autocorrelation but runs significantly slower than m1; m3 does not consider autocorrelation and instead adds date into random effects; m4 neither considers autocorrelation nor adds the date to random effects; m0 is consistent with m3, but estimation is done using the lme4 package. In fact, we found little difference in the estimation of fixed effects (Table S3). Comparing the AIC values among the four models still shows that m3 has a better fit (Table S4). Therefore, we believe our previous analysis is feasible.

| Table S2. Settings of four models | | | | | | | | | | | | |
| --- | --- | --- | --- | --- | --- | --- | --- | --- | --- | --- | --- | --- |
|  | Fixed | | | Random | | | | Correlation | | | | |
| m1 | Site * Distance | | | 1 \| Region | | | | AR( 1 \| Region) | | | | |
| m2 | Site * Distance | | | 1 \| Region / Plot | | | | AR( DateDay \| Region / Plot) | | | | |
| m3 | Site * Distance | | | 1 \| DateDay / Region | | | | NULL | | | | |
| m4 | Site * Distance | | | 1 \| Region | | | | NULL | | | | |
| Table S3. Comparison of estimates of fixed effect across models. | | | | | | | | | | | | |
|  | | m0 | | | m1 | | m2 | | | m3 | | m4 |
| Site | | -3.61 | | | -3.65 | | -3.60 | | | -3.61 | | -3.61 |
| SiteCF | | 0.76 | | | 0.81 | | 0.75 | | | 0.76 | | 0.76 |
| SiteTF | | -2.06 | | | -2.03 | | -2.06 | | | -2.06 | | -2.06 |
| Distance | | -0.19 | | | -0.17 | | -0.18 | | | -0.19 | | -0.19 |
| Dis:CF | | -0.14 | | | -0.16 | | -0.14 | | | -0.14 | | -0.14 |
| Dis:TF | | -0.08 | | | -0.09 | | -0.08 | | | -0.08 | | -0.08 |
| Table S4. Comparison of four models. | | | | | | | | | | | | |
|  | | | df | | | AIC | | | BIC | | loglik | |
| m1 | | | 9 | | | 11697 | | | 11757 | | -5839 | |
| m2 | | | 10 | | | 17010 | | | 17076 | | -8495 | |
| m3 | | | 9 | | | 8990 | | | 9050 | | -4486 | |
| m4 | | | 8 | | | 18649 | | | 18701 | | -9316 | |

-It’s stated that sensors “effectively avoid direct solar radiation”. This needs elaboration, such as what shielding was used? Were sensors inter-calibrated? What’s the margin of error?

Perhaps include calibration protocol, make/model of sensors, and specifications on shielding.

Response: In the revised manuscript, additional details about the sensor model and a supplementary explanation of the radiation shielding method have been provided in **Section 2.2** (**lines 156–159**). To validate the effectiveness of the shielding approach, a 30-day field test was conducted at Yunnan University’s Chenggong Campus in Kunming, Yunnan Province. Results indicated that the shielding method reduced surface temperature readings by approximately 1 °C under direct sunlight (S1 Fig).

S1 Fig. Diurnal variation of shading effect.

**Material and methods Section 2.2**, lines **156-159:**

A temperature sensor (HOBO® MX2301A) was mounted at 1.5m above the ground on each pole (Fig 1c), with a protective PVC pipe (diameter: 100mm, height: 10cm) shield to prevent direct sunlight exposure and thereby reduce the influence of solar radiation on temperature measurements [44].

-Day/night was classified using monthly averages of sunrise/sunset. This may be coarse for fine-scale hourly temperature analyses, especially around dawn/dusk or in areas with significant elevation change. I think hourly classification using exact date/time (e.g., daily ephemerides) would improve accuracy.

Response: In high-latitude regions, sunrise and sunset times change more rapidly than in low-latitude regions. To assess the potential impact of this variation, we examined the latitudinal range of our study. The highest latitude included in this study does not exceed 28°N. We recalculated the sunrise and sunset times throughout the year at 28°N and found that the maximum monthly variation is approximately 30 minutes. Given our hourly temporal resolution, grouping data by monthly averages remains appropriate.

The calculated sunrise and sunset times are as follows:

| **Date** | **Sunrise** | **Sunset** |
| --- | --- | --- |
| **2024-01-01** | **08:11** | **18:35** |
| **2024-02-01** | **08:07** | **19:00** |
| **2024-03-01** | **07:44** | **19:20** |
| **2024-04-01** | **07:10** | **19:37** |
| **2024-05-01** | **06:40** | **19:54** |
| **2024-06-01** | **06:24** | **20:12** |
| **2024-07-01** | **06:27** | **20:20** |
| **2024-08-01** | **06:42** | **20:09** |
| **2024-09-01** | **06:59** | **19:40** |
| **2024-10-01** | **07:12** | **19:05** |
| **2024-11-01** | **07:31** | **18:35** |
| **2024-12-01** | **07:54** | **18:23** |
| **2025-01-01** | **08:11** | **18:35** |

-Statements such as 270-271 “this represents the upper limit of cooling that forests can offer in response to macroclimatic warming” may be overreaching without modeling future climate interactions or including multiple years of data.

Response: Thank you for the valuable feedback from the reviewer. We have reorganized and added qualifiers to the presentation of the results in the revised manuscript in **Section 3.3** (**lines 303-305**).

**Results Section 3.3**, lines **303-305:**

This represents the upper limit of cooling exhibited by these forests in response to macroclimatic warming during the study period.

-Figures are generally clear, but some (e.g., Table 2) are dense and could be reformatted for readability. Including schematic diagrams of the sampling design in the main text would aid comprehension.

Response: Thank you for the reviewer’s meticulous guidance. In the revised manuscript, we have modified the format of Table 2 to improve clarity and readability. In addition, the description of the study area in **Section 2.1** (**line** **140**) has been updated to align with revisions made to Figure 1. Specifically, latitude and longitude lines were added to enhance geographic orientation, font sizes were adjusted for improved legibility, and forest types were labeled using the abbreviations TF, SF, and CF to maintain consistency with the rest of the manuscript. Lastly, we have revised the description of non-forest areas to provide greater accuracy and clarity.

**Fig 1. Map of the study region and details on instrument deployment.** (a) climate zone and vegetation types of the study area. (b) design of transect and quadrat for each forest region. Patch A represents forest patches, while Open area represents adjacent non-forest patches. The numbers 0 to 5 indicate 3 m × 3 m quadrats. (c) installation photos of the temperature monitor.

| **Table 2. The spatiotemporal variation of DEI and maximum cooling in natural forest type under multiple climatic backgrounds.** | | | | | | | | | | | | |
| --- | --- | --- | --- | --- | --- | --- | --- | --- | --- | --- | --- | --- |
| **DEI (MAT (°C))** | **Mar** | **Apr** | **May** | **Jun** | **Jul** | **Aug** | **Sep** | **Oct** | **Nov** | **Dec** | **Jan** | **Feb** |
|  |  |  |  |  |  |  |  |  |  |  |  |  |
| **CF** | 72(-4.2) | 62(-4.6) | 18(-5.4) | 19(-4.3) | 24(-5.2) | 39(-5.7) | 52(-5.7) | 64(-4.0) | 56(-5.8) | 73(-5.2) | 77(-4.5) | 66(-4.2) |
| **SF** | 65(-5.8) | 64(-4.9) | 46(-6.1) | 13(-5.9) | 15(-7.0) | 28(-6.3) | 47(-5.7) | 16(-5.5) | 41(-5.7) | 20(-4.2) | 45(-4.7) | 58(-5.3) |
| **TF** | 59(-6.3) | —— | —— | 17(-6.9) | 32(-7.5) | 46(-7.7) | 40(-7.8) | 48(-8.2) | 42(-7.6) | 55(-7.9) | 52(-7.7) | 50(-7.0) |
| **CF(S)** | 66(-4.6) | | | 40(-5.4) | | | 71(-5.2) | | | 80(-4.8) | | |
| **SF(S)** | 72(-5.7) | | | 35(-6.5) | | | 53(-5.7) | | | 57(-4.8) | | |
| **TF(S)** | 29(-6.3) | | | 40(-7.6) | | | 61(-7.9) | | | 58(-7.6) | | |
| **Whole year** | CF = 82(-5.0) SF = 72(-5.7) TF = 56(-7.5) | | | | | | | | | | | |
| The values in parentheses represent the maximum air temperature offset (MAT, °C) in the forest edge decoupling zone. "——" indicates that the DEI could not be quantified from the data. S represents the DEI and MAT of different forests in each season. | | | | | | | | | | | | |

-The discussion could briefly address potential implications under future climate change scenarios.

Response: Thank you for your thoughtful suggestion. In the revised manuscript, we have integrated our research findings into broader ecological theories in the **Discussion sections 4.1** (**lines 352-364**) and **4.1** (**lines 393-397**), including ecological niche dynamics and community assembly processes. In particular, we discuss how the forest edge buffering of microclimates can influence thermal niches, potentially moderating species persistence and shaping community structure under climate change.

**Discussion Section 4.1**, lines **351-363:**

These findings highlight the diverse thermal tolerances of plant species across climatic zones [57], with differential heat tolerance influencing habitat selection and driving distributional shifts toward warmer microclimates [58]. Microclimates delineate the realized thermal niches of forest plant species [59]. As microclimatic temperatures decline toward forest edges, species with varying thermal requirements across life stages may benefit [60], shaping their thermal preferences and habitat selection. These processes critically influence species composition and spatial distribution, as observed in birds [61] and insects [62,63]. Overall, our findings support the hypothesis that forest edges across climatic gradients exhibit distinct cooling patterns. The temperature offset in forests across different climatic zones was markedly distinct, with edge areas exhibiting increased cooling in warmer regions. The cooling thresholds for TF, SF, and CF were 8.2°C, 7.0°C, and 5.8°C, respectively. Edge-induced temperature effects vary across space and seasons among forest types along climatic gradients. These findings align with the ecological niche dynamics theory, suggesting that species persistence is shaped by localized thermal environments within broader ecological contexts.

**Discussion Section 4.2**, lines **393-397:**

Through monthly assessments of DEI, we revealed pronounced seasonal and regional variations, highlighting how forest types and climatic regimes shape the spatial extent of edge-induced microclimatic shifts. These findings suggest that edge-mediated microenvironments may function as ecological filters, influencing community assembly processes and supporting the identification of microrefugia, particularly for temperature-sensitive species [59,74].

Minor comments:

Line 55: Leave the citations for the end of the sentence to improve readability.

Response: Thank you for your careful observation. In the revised manuscript, the citations have been placed at the end of the sentence to enhance readability.

Line 117-118: scientific names should be italicized.

Response: Thank you for your attention to detail. In the revised manuscript, all scientific names (genus and species) mentioned in **Section 2.1 (lines 128, 132–133, and 136)** of the Materials and Methods have been italicized accordingly.

**Material and methods Section 2.1**, line **128:**

…including *Phoebe lanceolate* (Wall. ex Nees) Nees and *Pittosporopsis kerrii* Craib, among others.

lines **132-133:** such as *Eriobotrya bengalensis* (Roxb.) Hook. f., *Ilex szechwanensis* Loes. and *Ilex corallina* Franch.

lines **136:** The forest is also bordered by grassland, primarily dominated by *Abies georgei*.

Line 121: ditto

Response: Thank you for your attention to detail. In the revised manuscript, all scientific names (genus and species) mentioned in **Section 2.1 (lines 128, 132–133, and 136) of the Materials and Methods** have been italicized accordingly.

**Material and methods Section 2.1**, line **128:**

…including *Phoebe lanceolate* (Wall. ex Nees) Nees and *Pittosporopsis kerrii* Craib, among others.

lines **132-133:** such as *Eriobotrya bengalensis* (Roxb.) Hook. f., *Ilex szechwanensis* Loes. and *Ilex corallina* Franch.

lines **136:** The forest is also bordered by grassland, primarily dominated by *Abies georgei*.

Line 140: You mentioned existing studies, but you only cited one study.

Response: A citation to relevant literature employing similar experimental designs has been added in **Section 2.2** (**line** **153**) of the revised manuscript to support the methodological approach.

**Material and methods Section 2.2**, line **153:**

In a design similarly to existing studies [5,19], ……

Line 271-274: Proof-read, repetitions included.

Response: Thank you for your careful observation. In the revised manuscript, the sentence in **Section 3.3** (**lines** **305–307**) has been carefully revised, and redundant content has been removed to improve clarity and conciseness.

**Results Section 3.3**, lines **305–307:**

Our results demonstrate that identifying the effective interior of forests enhances understanding of the maximum cooling, which occurs at the monthly scale, however, to obtain the upper limit of cooling, finer time scales are necessary.

**References**

1. Kuznetsova A, Brockhoff PB, Christensen RHB. lmerTest Package: Tests in Linear Mixed Effects Models. J Stat Softw. 2017;82: 1–26. doi:10.18637/jss.v082.i13

2. Whittingham MJ, Stephens PA, Bradbury RB, Freckleton RP. Why do we still use stepwise modelling in ecology and behaviour? J Anim Ecol. 2006;75: 1182–1189. doi:10.1111/j.1365-2656.2006.01141.x

3. Meeussen C, Govaert S, Vanneste T, Bollmann K, Brunet J, Calders K, et al. Microclimatic edge-to-interior gradients of European deciduous forests. Agric For Meteorol. 2021;311: 108699. doi:10.1016/j.agrformet.2021.108699

4. Ouyang Z, Liu K, Lu M, Ouyang Z, Liu K, Lu M. Bias correction based on AR model in spurious regression. AIMS Math. 2024;9: 8439–8460. doi:10.3934/math.2024410

5. Davis LA. Estimating random effects and serial correlation in random coefficient models: to weight or not to weight. 2002. Available: http://hdl.handle.net/1807/122886

6. Serial correlation or random subject effects. Commun Stat - Simul Comput. 1990 [cited 12 Jun 2025]. doi:10.1080/03610919008812907
