# Supplementary material for: Spatiotemporal variation of the maximum cooling effect across edge-to-interior gradients in forest patches of southwestern China
Source: PLoS One. 2026 Feb 12;21(2):e0342179. doi: 10.1371/journal.pone.0342179 (PMC12900313; doi:10.1371/journal.pone.0342179)
Supplement: Attachment — Submitted filename: PONE-D-25-08797_Review2_v1.docx [file pone.0342179.s004.docx]

Spatiotemporal variation of the maximum cooling effects across edge-to-interior gradients in natural forest patches of Southwest China

**Summary**:

It appears the authors have provided a revised manuscript but failed to update the line numbers to accurately pinpoint their edits via responses to reviewer comments. They have, however, provided those edits in full to specific reviewer comments. Overall, the authors have done a decent job addressing reviewer concerns and comments. I have provided some edits below to improve the manuscript.

Edits:

**Title**:

- The authors should ensure that “Southwest China” is being used correctly. Southwest China is a much larger area than the study area. This appears to be the Yunnan Administrative Division. The use of “southwest China” is more accurate as it describes a general area.
- Remove “natural” in title or replace with unmanaged. Natural here implies there are unnatural forest patches. I think the authors mean unmanaged as natural?

**Abstract**:

Line 26: it is not that it has received limited attention or “remains insufficiently understood” (Line 24), rather it is an active area of inquest where we are still trying to understand patterns and determine how those patterns link to ecological effects and generality to form theory.

Line 36-39: the Abstract ends with linkages to how this research may contribute to management strategies but does not provide those “insights”. In the Conclusion section the authors briefly re-iterate this messaging but, again, do not provide tangible strategies of how this work could contribute to management i.e., climate refugia modelling, landscape connectivity by defining thermal effects of edges or parameterization of thermal resistance scores…etc. Some elaboration on these management related links would strengthen the manuscript greatly and make it more tangible for land managers.

**Methods**:

Line 128-129: Instead of testing the “expected exponential decay” by having regular spaced samples they leaned on Zellwegers study to inform their sampling design and consequently cannot say with spatial accuracy where thresholds of thermal inflection occur. If temperature buffering of thermal edges is “insufficiently understood” as noted in the Abstract, then why use a study to inform the sampling design of this work? The authors need to emphasize and thoroughly demonstrate why this approach was used. I highlighted this in my previous review. This is also a limitation of their study and should go in section 4.3

- Response to one of my questions
-

  - Do these forests of minimal anthropogenic or natural disturbance reflect a realistic population of forest patches in these ecosystems? Or are these patches a rarity and thus less applicable to informing management and ecological theory in the anthropocene.
  - I can understand the need to minimize anthropogenic effects for study control purposes but natural disturbances occur and should occur in fact the lack of natural disturbances may be due to anthropogenic effects in adjacent areas i.e., forest health management of insects or pathogens.

**Discussion**:

Line 324-325: The realized niche is the product of abiotic and biotic interactions. More accurately, microclimates fine tune our estimates of fundamental niche effects. The authors also need to consider the scales of habitat selection 1^st^, 2^nd^, 3^rd^ and 4^th^ order effects (Johnson 1980, Ecology) and which scale micro and macro climate act on a given species as it interacts with niche processes.

Line 334: reference? I don’t think the authors have accurately demonstrated how their findings align with ecological niche theory? I don’t know what ecological niche “dynamics” theory is… it is not a thing… “dynamics” should be removed.

Line 369: Remove “major” these are simply limitations.

Line 378: the authors need to demonstrate why horizontal and vertical transects are being recommended here.
