# Supplementary material for: Spatiotemporal variation of the maximum cooling effect across edge-to-interior gradients in forest patches of southwestern China
Source: PLoS One. 2026 Feb 12;21(2):e0342179. doi: 10.1371/journal.pone.0342179 (PMC12900313; doi:10.1371/journal.pone.0342179)
Supplement: Attachment — Submitted filename: 05_Response to Reviewer1.docx [file pone.0342179.s006.docx]

Dear Editors and reviewer 1:

We sincerely thank Reviewer #1 for the careful evaluation of our manuscript and for providing valuable comments that have greatly helped us improve the clarity, robustness, and broader relevance of our study. We have carefully considered each comment and revised the manuscript accordingly. We have addressed the reviewer’s key concerns regarding:

*1. The representativeness and robustness of the dataset (sample size, transect replication, and temporal coverage).*

*2. The inclusion and discussion of forest structural variables (e.g., canopy height, cover, and density).*

*3. The generalizability and broader ecological implications of our findings.*

*Detailed point-by-point responses are provided below.*

**Comment 1:**

The representativeness and robustness of the dataset (sample size, transect replication, and temporal coverage).

**Response:**

We sincerely appreciate this valuable and constructive suggestion. We fully recognize that relying on one transect per forest type and one year of data may restrict the generalizability of our findings. To evaluate this concern empirically and to assess whether our transect design can adequately represent the spatiotemporal variability of forest microclimates, we conducted an independent replication snapshot this year near each original transect.

(1) Field design.

For each forest type, we established four additional parallel transects located within the same stand and environmental context as the original transect. Each new transect contained five sampling points positioned at the same distances from the forest edge as those used in the original study (1.5, 4.5, 12.5, 36.5, and 99.5 m). Together with the original transect, this resulted in 25 observation points per forest type. All temperature measurements were taken at a height of 1.5 m above ground, consistent with the setup used for the automatic sensors.

(2) Measurements.

At each point, air temperature was recorded for approximately 30 minutes using portable, shielded temperature loggers (accuracy ±0.2 °C). The measurement time window was synchronized with the concurrent 10-minute records from the open reference and the automatic sensors. For each location, the median value of the 30-minute measurement period was used to minimize short-term fluctuations in temperature.

(3) Analytical verification.

The supplementary dataset was analyzed using the same linear mixed-effects modeling framework as described in the manuscript (log-distance and forest type as fixed effects; date nested within site as random intercept). The results confirmed that the edge-to-interior cooling gradient and maximum cooling intensity from the new transects were consistent in direction and magnitude with those derived from the continuous automatic monitoring. The estimated distance of edge influence (DEI) values likewise fell within the 95 % confidence intervals of the original estimates.

(4) Supplementary verification figure.

To visualize this consistency, the replication snapshot data were integrated into an updated model (S4 Fig.). The new analysis demonstrates that including the parallel transects does not alter the overall shape or magnitude of the predicted temperature offset. The colored curves in Fig. 1 represent forest types across climatic zones (CF = temperate coniferous, SF = subtropical evergreen broadleaf, TF = tropical forest), and the gray ribbons denote 95 % confidence intervals. The stable overlap of the curves confirms the robustness of the edge-to-interior temperature gradient after the supplementary data were incorporated.

(5) Conclusion.

These findings indicate that the transects established in our study reliably capture the relative spatial patterns and magnitudes of microclimatic variation across the three representative forest types, within the temporal and spatial scope of the current design.

We have inserted explicit cross‐references to the parallel snapshot transects in Sections 2.2, 2.3.2, 3.1, and 4.1 of the main text, added S4 Fig and Table S1 to the Supporting information list, and acknowledged the single‐year coverage in Section 4.3 as an additional limitation. These changes align the manuscript with our point‐by‐point response and address the reviewer’s concerns about replication and robustness.

S4 Fig. Model predictions of the air-temperature offset (°C) as a function of distance from the forest edge (m), combining the original automatic monitoring data and the new snapshot-transect measurements. Colored lines indicate natural forest types in different climatic zones (CF = temperate coniferous forest, SF = subtropical evergreen broadleaf forest, TF = tropical forest). Gray shaded ribbons show 95 % confidence intervals. Point transparency and jittering were applied to improve visibility.

**Comment 2:**

The inclusion and discussion of forest structural variables (e.g., canopy height, cover, and density).

**Response:**

We sincerely thank the reviewer for this valuable suggestion. As mentioned in the previous response, the supplementary field campaign also allowed us to evaluate how stand structural attributes influence the spatial pattern of the forest–edge microclimate. At each monitoring point—both the original automatic sensors and the newly established snapshot transects—we measured three representative structural variables that capture different aspects of forest architecture:

- BA (basal area, m² ha⁻¹): quantifies stand density and stem dominance.
- SC (shrub cover, %): indicates understorey density and near-ground shading.
- THm (mean tree height, cm): reflects overall canopy height and vertical structure.

We incorporated these variables as fixed effects and tested their interaction with distance from the forest edge using the same linear mixed-effects framework employed in the main analysis. In the supplementary structural analysis (Table S1), basal area (BA) showed a negative main effect on air-temperature offset across forest types (denser stands = more negative Toffset, i.e., stronger cooling), whereas the main effect of shrub cover (SC) was weak/positive overall and varied by forest type. Crucially, Distance × SC interactions were negative and significant in CF and TF, indicating that higher understorey cover steepens the edge-to-interior cooling gradient; by contrast, Distance × BA interactions were positive and significant in CF and SF, suggesting that BA-related cooling attenuates with distance from the edge. THm was non-significant. These results support the view that forest structure modulates edge microclimates, with understorey cover strengthening the distance-dependent cooling, while basal area exerts stronger effects near edges (See Table S1 and Fig. S4.).These findings emphasize that, beyond large-scale climatic context, local forest structure plays an additional regulatory role in shaping microclimate heterogeneity near forest edges.

We have (i) added Methods text describing the supplementary structural-covariate analysis (BA, SC, THm) and its rationale, (ii) summarized key structural effects in Results, (iii) revised Section 4.1 to replace the previous statement that mechanisms were “not directly quantified” with a paragraph reporting the Table S1 findings (negative BA main effect; negative Distance × SC in CF/TF; positive Distance × BA in CF/SF), and (iv) updated the Supporting information list to include Fig. S4 and Table S1. These changes align the manuscript with our response and remove potential inconsistencies.

| **Table S1.** Effects of stand structural variables in different forest types (BA = basal area; SC = shrub cover; THm = mean tree height), distance to the forest edge, and their interaction effects on air-temperature offset (°C). Coefficient estimates are shown; significance is indicated by asterisks (* p < 0.05, ** p < 0.01, *** p < 0.001). | | | |
| --- | --- | --- | --- |
| **Air temperature offset (°C)** | **CF** | **SF** | **TF** |
| **Intercept** | -1.63** | -1.76** | -3.75*** |
| **Distance to the edge  (log-transformed, m)** | -5.14×10^-1^** | -8.39×10^-1^*** | -7.42×10^-1^* |
| **BAs** | -1.20×10^2^ | -3.29×10^2^ | -2.31×10^3^ |
| **SC** | 8.99×10^-3^ | 5.17×10^-3^ | 7.13×10^-2^* |
| **THm** | -1.15×10^-3^ | -3.40×10^-3^ | -6.85×10^-3^ |
| **Distance: BAs** | 1.02×10^2^ | 3.96×10^2^ | 1.04×10^3^* |
| **Distance: SC** | -7.27×10^-2^* | -3.23×10^-3^ | -2.73×10^-2^* |
| **Distance: THm** | -9.89×10^-3^ | 1.16×10^-3^ | 1.53×10^-3^ |

**Comment 3:**

The generalizability and broader ecological implications of our findings.

**Response:**

We fully agree with the reviewer’s insightful comments. As described above, we have already initiated additional replication and structural measurements following the reviewer’s valuable suggestions. The transect monitoring introduced in this manuscript represents part of a long-term observation program that continues to document forest-edge microclimate dynamics in Southwest China.

We acknowledge that broader generalization will require multi-year data and greater spatial replication; nonetheless, our present dataset provides a rare and regionally significant baseline for understanding microclimate variation in this highly fragmented mountain landscape. Because empirical microclimate data remain scarce across this region, we consider the reporting of these baseline edge-to-interior patterns an important step toward filling a global data gap and enabling parameterization and validation of advanced microclimate mechanism models.

Furthermore, this work contributes to a better ecological understanding of how forest fragmentation and canopy heterogeneity modulate near-ground thermal environments. The results offer essential references for forest-climate modeling, biodiversity assessments, and conservation planning in topographically complex tropical and subtropical regions.

In future work, we will build on these foundations by applying UAV-LiDAR and 3-D structural modeling approaches to quantify canopy architecture and its role in microclimate regulation, as well as by extending the monitoring network to multi-year observations for stronger spatial and temporal inference.
