# Supplementary material for: Spatiotemporal variation of the maximum cooling effect across edge-to-interior gradients in forest patches of southwestern China
Source: PLoS One. 2026 Feb 12;21(2):e0342179. doi: 10.1371/journal.pone.0342179 (PMC12900313; doi:10.1371/journal.pone.0342179)
Supplement: Attachment — Submitted filename: 05_Response_to_Reviewer1_auresp_3.docx [file pone.0342179.s007.docx]

Dear Editors and reviewer 1:

We sincerely thank Reviewer #1 for the careful evaluation of our manuscript and for providing valuable comments, which have greatly helped us improve the clarity, robustness, and broader relevance of our study. We have carefully considered each comment and revised the manuscript accordingly. We have primarily addressed the reviewer’s key concerns regarding:

*1. Provide a clearer mechanistic explanation for why cooling differs among forest types (beyond reporting magnitudes). Add key canopy structural measurements (such as canopy cover/closure/openness from hemispherical photographs) to support the interpretation.*

*2. Strengthen the practical implications in the Introduction and Discussion, linking edge-to-interior thermal dynamics to forest management and species adaptation, and add specific ecological examples (e.g., understory biodiversity).*

*3. Add details on the snapshot replication timing and temporal coverage (dates and sampling duration).*

*4. Clarify and adopt more cautious terminology, such as “long-term” and “high-precision”.*

**Comment 1:**

Strengthened mechanistic interpretation of the observed cooling differences among forest types by incorporating an expanded dataset, including newly collected understory temperature measurements with additional transect replication, stand/structural variables, and canopy structural metrics derived from hemispherical photographs (canopy openness, Openness, and leaf area index, LAI).

**Response:**

We agree with the reviewer that reporting cooling magnitudes alone is not sufficient without mechanistic interpretation. To address this, we expanded our dataset by adding forest structural metrics, including **canopy openness (Openness) and leaf area index (LAI) derived from hemispherical photographs**, together with additional stand/structural variables. We also incorporated **short-term replicated transect temperature measurements** to strengthen spatial support for the observed edge-to-interior patterns. Based on these additions, we provide an initial, structure-informed explanation for why cooling intensity differs among forest types. Corresponding revisions have been made in the **Materials and Methods, Results, and Discussion**, and we have updated the **Abstract and Conclusions** to reflect the enhanced mechanistic interpretation. We also explicitly acknowledge the remaining limitations and outline how future work (e.g., broader replication and concurrent process measurements) can further improve mechanistic attribution.

**Materials and Methods**

To assess whether the year-long automated transect captured spatially representative edge-to-interior patterns within each forest, we established four additional transects parallel to the primary transect for snapshot measurements. This resulted in five transects per forest when including the original transect, and the snapshot design is described in the Supplementary Material.

Spatial representativeness of the automated transect was evaluated using snapshot transects. The log-distance mixed model was refitted to the combined dataset that included both the year-long automated transect and snapshot measurements. Predicted edge-to-interior curves overlapped and remained within the 95% confidence intervals, indicating that the automated transect captured a spatially consistent gradient within each site (S4 Fig). Snapshot transects were collected in a different year and within a limited measurement window, so they were used only to assess spatial robustness and were not used to infer temporal dynamics.

**Results**

Spatial robustness was supported by snapshot transects. Snapshot derived offsets aligned with the automated transect predictions and remained within the 95% confidence bands, indicating that the automated transect captured a consistent edge to interior pattern within each site (S4 Fig). Supplementary structure analyses suggested that the distance response can be modulated by canopy structure in a site-specific manner, with significant negative interactions between log distance and LAI in the temperate and subtropical sites and between log distance and canopy openness in the temperate site (S1 Table).

**Discussion**

Supplementary structure analyses provide additional insight into how the edge gradient in maximum cooling can be shaped locally. Distance interactions with LAI were negative in the temperate coniferous and subtropical sites, and a negative distance interaction with canopy openness was detected in the temperate coniferous site (S1 Table). These results suggest that spatial changes in canopy density and openness along the transect can modulate how rapidly cooling strengthens from the edge to the interior [16,36,43,54]. Denser canopies can reduce radiative inputs and damp air exchange with the surrounding matrix, which promotes faster accumulation of cooling with distance [43]. More open edges can increase shortwave penetration and turbulent mixing, which weakens cooling near the boundary and delays the emergence of interior like conditions [16,29,55]. Although these structural signals were forest specific, they align with established mechanisms linking canopy structure and mixing to microclimate buffering [36,55,56].

| **S1 Table. GAM results for stand structural variables in different forest types (BA = basal area; SC = shrub cover; THm = mean tree height; Openness = canopy openness; LAI = leaf area index), including key linear interaction terms with log-distance (Distance: Openness and Distance: LAI) and a distance-to-edge smooth on air-temperature offset (C).** | | | |
| --- | --- | --- | --- |
| **Variable** | **CF** | **SF** | **TF** |
| **Intercept** | -3.326^***^ | -2.997^***^ | -5.121^***^ |
| **s(Distance_log)** | 2.24 (7.31)^***^ | 1.00 (27.96)^***^ | 2.52 (34.11)^***^ |
| **BA** | -83.325 | 1.11×10^-3^ | -2.36×10^2^ |
| **SC** | -4.39×10^-3^ | -8.04×10^-3^ | 4.27×10^-3^ |
| **THm** | 2.16×10^-3^ | -1.56×10^-3^ | 4.69×10^-3^ |
| **Openness** | -0.043 | -0.090 | -0.024 |
| **LAI** | -0.731 | -0.759^**^ | 0.112 |
| **Distance:Openness** | -0.067^*^ | -0.041 | -4.39×10^-3^ |
| **Distance:LAI** | -0.768^*^ | -0.289* | 0.155 |
| Continuous predictors were mean-centered before forming interaction terms; Openness/LAI collinearity was evaluated via mean-centering and sensitivity models. Parametric coefficients are shown for linear terms, and smooth terms are reported as edf (F). Significance is indicated by asterisks (* p < 0.05, ** p < 0.01, *** p < 0.001). | | | |

**S4 Fig. Model predictions of the air-temperature offset (C) as a function of distance from the forest edge (m), combining the original automatic monitoring data and the new snapshot-transect measurements.** Colored lines indicate natural forest types in different climatic zones (CF = temperate coniferous forest, SF = subtropical evergreen broadleaf forest, TF = tropical forest). Gray shaded ribbons show 95 % confidence intervals. Point transparency and jittering were applied to improve visibility.

**Comment 2:**

Strengthen the practical implications in the Introduction and Discussion, linking edge-to-interior thermal dynamics to forest management and species adaptation, and add specific ecological examples (e.g., understory biodiversity).

**Response:**

Thank you for this helpful comment. We agree that the practical relevance of edge-to-interior thermal dynamics should be more explicitly highlighted. Accordingly, we have reorganized and revised both the **Introduction** and **Discussion** to strengthen the management and species-adaptation implications of our findings, with particular emphasis on potential consequences for understory biodiversity. In addition, we added a dedicated subsection in the **Discussion** (**Section 4.3)** that synthesizes how DEI-based delineation of thermally decoupled interiors can inform the identification of microrefugia and support edge-aware management decisions.

**Discussion Section 4.3 DEI-defined decoupled interiors as microrefugia and management implications**

DEI-based delineation of coupled and decoupled zones provides a practical way to define microrefugia using thermal decoupling rather than a fixed distance alone. Across sites, decoupled interiors consistently exhibited stronger extreme cooling than coupled edge zones, which supports their functional interpretation as daytime thermal refugia under high heat load [58,59]. Cooling magnitudes in the decoupled interior were substantial and reached approximately 8°C in the tropical site. Such differences are large enough to shift thermal safety margins for temperature-sensitive understory organisms and to alter the conditions experienced during short-lived heat events [68,69].

Microrefugia defined in this way are inherently dynamic. Both the extent and, in some months, the presence of decoupled interiors varied strongly through the year. In the tropical site, a decoupled interior was not detected within 99.5 m during April and May. This pattern is consistent with a seasonal contraction of thermally decoupled interiors or with a DEI that exceeded the sampled transect [29,57]. Either interpretation implies reduced refuge availability during a period that can coincide with high thermal stress in the pre-monsoon season. Seasonal variability in canopy energy exchange offers a coherent explanation. Shifts in cloudiness, evaporative demand, and water availability can change the temperature contrast between open and forest environments and can modify how rapidly interior-like conditions emerge with distance from the edge [12,44,70].

This decoupling framework has direct management relevance because it translates microrefugia into a measurable spatial threshold. A forest patch can only provide a DEI-defined refugium if it contains area where the distance to the nearest non-forest edge exceeds DEI. When the maximum distance to edge within a patch is smaller than the relevant DEI, a thermally decoupled interior is unlikely to form, and the patch may fail to sustain the strongest cooling function even if mean offsets remain negative. This provides a clear basis for evaluating fragmentation impacts and for prioritising patch retention [35,71]. Distance-to-edge maps can be combined with DEI estimates to quantify the fraction of each patch that qualifies as potential refugial core under specific months or seasons [72].

Conservative design for year-round buffering can be based on the largest DEI observed across seasons or across critical months, whereas climate-adaptive planning can target DEI values for the periods when heat stress risk is highest. This time-specific approach avoids relying on a single annual DEI that may obscure months when decoupled interiors contract [51,52]. Because each forest type was represented by one focal site, the numerical thresholds reported here should be interpreted as evidence from a climatic gradient rather than universal limits. Replication across multiple sites per forest type and concurrent measurements of radiation, wind, and moisture would further strengthen mechanistic attribution and improve transferability of DEI-based guidance for edge-aware conservation planning in fragmented landscapes [11,13,73].

**Comment 3:**

Add details on the snapshot replication timing and temporal coverage (dates and sampling duration).

**Response:**

Thank you for the reviewer’s comment. We have now added full details on the snapshot replication in the manuscript and Supplementary. The community-structure variables and understory air temperature data were collected from 1–25 April 2025. Each forest site was surveyed for at least one week during this period. Measurements were conducted between 09:30 and 18:00, and we visited all plots at least twice within this window, with each measurement lasting 20 minutes

**Comment 4:**

Clarify and adopt more cautious terminology, such as “long-term” and “high-precision”.

**Response:**

Thank you for the careful and constructive comment. We agree that the wording should be more precise. In the revised manuscript, we have replaced “long-term” with a more accurate description of the monitoring duration (i.e., one year of continuous monitoring) and we no longer refer to it as “long-term.” We also clarified that “high-precision” refers to the high temporal resolution and standardized deployment of the temperature loggers (e.g., consistent sensor type, placement height, radiation shielding, and logging interval) rather than implying unusually low measurement error. In addition, we have reorganized and partially rewritten the manuscript to improve terminology consistency and strengthen the logical coherence across sections.
