# Supplementary material for: Spatiotemporal variation of the maximum cooling effect across edge-to-interior gradients in forest patches of southwestern China
Source: PLoS One. 2026 Feb 12;21(2):e0342179. doi: 10.1371/journal.pone.0342179 (PMC12900313; doi:10.1371/journal.pone.0342179)
Supplement: Attachment — Submitted filename: 05_Response_to_Reviewer1_auresp_4.docx [file pone.0342179.s008.docx]

Dear Editors and reviewer 1:

We sincerely thank Reviewer #1 for the careful evaluation of our manuscript and for providing valuable comments. We have revised the manuscript accordingly and updated the line numbering throughout the tracked-changes version so that every edit can be located unambiguously. We have primarily addressed the reviewer’s key concerns regarding:

*1. Clarifying the relevant wording and explicitly noting in the main text that forest-structure measurements were conducted for the snapshot transects.*

*2. Streamlining the Discussion to avoid repeating Results.*

Detailed point-by-point responses are provided below.

**Comment 1:**

Line 97: These are not questions. Please revise for clarity.

**Response:**

Thank you for the careful comment. In the revised manuscript, we revised the relevant text by framing it explicitly as study objectives and introducing the three aims with “We address three aims:”, thereby improving clarity and consistency.

**Comment 2:**

Lines 160–161: The authors refer readers to the supplementary for details on the snapshots. However, they should also mention that forest structure measurements were conducted and are detailed in the supplementary (as some of those results are referenced in the discussion).

**Response:**

Thank you for this valuable comment. In the revised manuscript, we have added a description of the stand-structure measurements and hemispherical fisheye photography, and we clarify that the methods are provided in the Supplementary Material.

Materials and methods, Lines 160-164:

This resulted in five transects per forest when including the original transect. In addition, we conducted stand-structure measurements and took hemispherical fisheye photographs in all plots across the five transects to derive forest structural metrics, such as canopy openness (Openness) and leaf area index (LAI), and the snapshot design and methodological details are described in the Supplementary Material.

**Comment 3:**

L270-272: This is not a result, and repeats the same statement from lines 211-213.

**Response:**

Thank you for this valuable comment. We revised and de-duplicated the relevant text in the revised manuscript. In the Materials and Methods (Lines 214–215 in the revised manuscript; formerly Lines 211–213), we now describe only the snapshot-based procedure and criteria used to assess spatial representativeness, and we removed result-oriented statements. In the Results (Lines 272–273 in the revised manuscript; formerly Lines 270–272), we rewrote the text as objective result statements, avoided repeating the methods, and retained/clarified the additional findings from the supplementary structure analyses.

Materials and Methods, lines 214–215:

This fit was used to generate predicted edge-to-interior response curves and corresponding 95% confidence intervals for each site (S4 Fig).

Results, lines 272–273:

Snapshot derived offsets aligned with the automated transect predictions and remained within the 95% confidence bands across sites (S4 Fig).

**Comment 4:**

L371-373: Repeats the same statement of results from lines 326-329.

**Response:**

Thank you for this comment. In the revised manuscript, we removed the result-repeating numerical statements (including the peak-month listing) from the Discussion and retained only a brief qualitative statement.

Discussion, lines 371-372:

Seasonal timing also mattered, and the strongest decoupled interior cooling occurred in different months across sites (Table 2).

**Comment 5:**

L389-391: Repeats the same statement of results from lines 272-275.

**Response:**

Thank you for this comment. In the revised manuscript, we removed the repeated interaction statements from the Discussion and retained only a brief qualitative statement.

Discussion, lines 386-386:

Supplementary structure analyses suggest that local variation in canopy structure may influence how rapidly maximum cooling strengthens from the edge to the interior (S1 Table).

**Comment 6:**

L411-413: Repeats the same statement of results from lines 295, 435.

**Response:**

Thank you for the careful and constructive comment. In the revised manuscript, we removed this repeated result statement from the scale-dependence paragraph (Lines 411–413) and retained it only once where it is interpreted mechanistically (Lines 435 in the revised manuscript; formerly Lines 428-432).

Discussion, lines 428-432

In contrast, in TF during April–May, edge–interior coupling appears to be especially strong, such that DEI may extend beyond the sampled 99.5 m transect. This pattern is consistent with reduced canopy cooling capacity and/or enhanced coupling to the surrounding matrix [11,68,69], which could shift DEI beyond the sampled extent.

In addition to the revisions made in response to the reviewer comments, we also further streamlined the Discussion to improve clarity and avoid redundancy. Specifically, we revised the text formerly in Lines 452–454 (now Lines 447-449 in the revised manuscript) and removed overlapping wording that repeated statements already presented elsewhere.

Discussion, lines 447-449:

Microrefugia defined in this way are inherently dynamic. Both the extent and, in some months, even the presence of thermally decoupled interiors varied markedly through the year, indicating that refugial availability is time dependent.
